# Phenotypic frailty and multimorbidity are independent 18-year mortality risk indicators in older men: The Helsinki Businessmen Study (HBS)

**DOI:** 10.1007/s41999-021-00472-w

**Published:** 2021-03-04

**Authors:** Timo E. Strandberg, Linda Lindström, Satu Jyväkorpi, Annele Urtamo, Kaisu H. Pitkälä, Mika Kivimäki

**Affiliations:** 1 University of Helsinki, Clinicum, and Helsinki University Hospital, Haartmaninkatu 4, PO Box 340, 00029 Helsinki, Finland; 2 University of Helsinki, Helsinki, Finland; 3 Center for Life Course Health Research, University of Oulu, Oulu, Finland

**Keywords:** Disability, Frailty, Mortality, Multimorbidity, Prefrailty

## Abstract

**Purpose:**

Multimorbidity, prefrailty, and frailty are frequent in ageing populations, but their independent relationships to long-term prognosis in home-dwelling older people are not well recognised.

**Methods:**

In the Helsinki Businessmen Study (HBS) men with high socioeconomic status (born 1919–1934, *n* = 3490) have been followed-up from midlife. In 2000, multimorbidity (≥ 2 conditions), phenotypic prefrailty and frailty were determined in 1365 home-dwelling men with median age of 73 years). Disability was assessed as a possible confounder. 18-year mortality follow-up was established from registers and Cox regression used for analyses.

**Results:**

Of the men, 433 (31.7%) were nonfrail and without multimorbidity at baseline (reference group), 500 (36.6%) and 82 (6.0%) men had prefrailty or frailty, respectively, without multimorbidity, 84 (6.2%) men had multimorbidity only, and 201 (14.7%) and 65 (4.8%) men had prefrailty or frailty together with multimorbidity. Only 30 (2.2%) and 86 (6.3%) showed signs of ADL or mobility disability. In the fully adjusted analyses (including ADL disability, mental and cognitive status) of 18-year mortality, frailty without multimorbidity (hazard ratio 1.62, 95% confidence interval 1.13–2.31) was associated with similar mortality risk than multimorbidity without frailty (1.55, 1.17–2.06). The presence of both frailty and multimorbidity indicated a strong mortality risk (2.93, 2.10–4.07).

**Conclusion:**

Although multimorbidity is generally considered a substantial health problem, our long-term observational study emphasises that phenotypic frailty alone, independently of disability, may be associated with a similar risk, and a combination of multimorbidity and frailty is an especially strong predictor of mortality.

## Key summary points

**Objective:**

Multimorbidity, phenotypic prefrailty and frailty are frequent in ageing populations

**Findings:**

This long-term follow-up of home-dwelling older men reveals the relationship of phenotypic frailty to long-term prognosis, independently of the presence of significant chronic diseases and disability.

**Message:**

Assessment of phenotypic frailty and already prefrailty provides extra clinical value for the assessment of prognosis in old age.

## Introduction

Multimorbidity, is an increasingly frequent condition in ageing societies although its prevalence naturally depends upon the definition of the conditions counted [1–4]. The prevalence has two sharply rises, first one around 50–60 years of age when chronic diseases start to appear, and second one in older age. Consequently, multimorbidity is gaining increasing attention, because it complicates treatment leading to greater susceptibility to failures of care delivery and co-ordination. Multimorbidity is often associated with polypharmacy predisposing to potential drug interactions, and is overall connected with impaired prognosis.

Frailty, on the other hand, is a clinical geriatric syndrome with a prevalence of 10–12% among populations aged 70 years or older, the prevalence further increasing in older age groups [5, 6]. Frailty is characterised by increased vulnerability to inner and outer stressors and is a risk factor of disability, hospitalisation and death [6, 7]. Frailty still lacks a consensus definition, but the two most frequent ways to define frailty are the phenotype method [8] and the calculation of cumulative deficits or frailty index [9].

The relationships between multimorbidity and frailty are complex, but considered multidirectional: multimorbidity predisposes to frailty and vice-versa [10–15]. Disability has also been shown to predict mortality independently of multimorbidity [16]. Consequently, the relationship between multimorbidity, frailty and mortality is quite obvious, if frailty index of cumulative deficits is used, because the definition also includes various diseases and disabilities [9]. Therefore, frailty as a phenotype is of special interest, because it is not considered a disability state [5] and offers an additional method, independently of diagnosed diseases to predict mortality and outcome risk. Because there are few long-term studies on this, we explored their relationships during a 18-year follow-up in the longitudinal Helsinki Businessmen Study (HBS) [17]. At baseline, these older men were home-dwelling with low prevalence of disability.

## Material and methods

### Study overview

We report a secondary analysis of the Helsinki Businessmen Study (HBS), a cohort of men born between 1919 and 1934 (original *n* = 3490), who have been followed-up since the 1960s [17, 18]. Their cardiovascular disease risk factor history [including body mass index (BMI)] is known since midlife (mean age 40 years). In 2000, current residential addresses were retrieved from the Population Information System of Finland for surviving HBS participants, and a questionnaire survey was sent to them. The questionnaire included items about lifestyle (smoking, alcohol consumption, physical activity), social status (married/divorced/widowed/unmarried), body mass index (BMI), medications, prevalent physician-diagnosed diseases, question about subjective memory disturbances, and health-related quality of life (HRQoL, RAND-36/SF-36 instrument [https://www.rand.org/health/surveys_tools/mos/36-item-short-form.html, also including questions about mobility and Activities of Daily Living (ADL)] [19, 20].

### Study groups in 2000

There is currently no consensus definition of the frailty syndrome, and various methods can be found in the literature. In the HBS, we have defined frailty phenotype using mainly questionnaire data and according to a modification of the Cardiovascular Health Study (CHS) frailty phenotype [21]. The following criteria were used: (1) Weight loss defined as > 5% weight loss from baseline in 1974, or having current calculated BMI under 21 kg/m^2^ in 2000. (2) evaluation of physical weakness based on self-reported difficulty (not at all = 0) in carrying or lifting a grocery bag (one of the questions of the Physical Function scale of RAND-36). (3) assessment of exhaustion based on reported low energy most or all of the time during the preceding 4 weeks (one of the questions of the Vitality scale of RAND-36). (4) evaluation of physical activity based on a question: “do you exercise regularly weekly?” the answer “no” was taken to denote low physical activity. Responders with 3–4, 1–2, and zero criteria were classified as frail, prefrail, and nonfrail, respectively. We have earlier demonstrated prognostic validity of this modification in our cohort [21].

In the literature there is no unequivocal definition of multimorbidity either [22], often defined as the presence of 2 or more diseases or clinical conditions. In the present study, our objective was to compare the independent effects of frailty phenotype (not based on diagnosed diseases and disabilities) and clinically meaningful, diagnosed diseases on mortality. Therefore, we defined multimorbidity as the reported presence of at least 2 out of 8 important chronic disease states: coronary artery disease (chronic or history of myocardial infarction), stroke, peripheral artery disease, heart failure, chronic pulmonary disease (including asthma and chronic obstructive pulmonary disease), diabetes (type not defined, but majority likely type 2), any cancer and musculoskeletal disease (including rheumatic diseases and osteoarthritis). We intentionally did not include hypertension, because it can be considered a risk factor rather than a disease. There were few cases of reported diagnosed psychiatric disorders (*n* = 55) or dementia (*n* = 6) among responders. Therefore, we used the Mental Health domain of RAND-36 and self-report of memory disturbances (‘Have you had problems with your memory?’) as proxies to reflect psychological and cognitive status, respectively.

We studied disability as a potential confounder. Using RAND-36 items, mobility disability was defined as ‘at least a lot of difficulty in walking ½ kilometer’ or ‘at least a lot of difficulty in climbing one flight of stairs’ [23], and ADL disability was defined as ‘a lot of difficulty in bathing or dressing oneself’. Smoking was categorised as current vs ex- or never smoker or as reported number of smoking years. Current and midlife BMI was calculated from the formula: weight in kg/height in m^2^. Alcohol consumption (grams of ethanol per week) was derived from responses to questions about participant’s consumption of different alcohol beverages in an average week.

### Outcome

Total mortality of the whole HBS cohort up to 31 March 2018 was retrieved from the Finnish Population Information System, which is a registry of all Finnish citizens. The register's assessment of vital status is reliable for people who permanently reside in Finland (over 95% of the present cohort), irrespective of whether they die in Finland or abroad. Moreover, the assessment of vital status is also quite reliable for Finnish citizens living permanently abroad.

### Statistical analysis

We used descriptive statistics, Armitage test for trend in proportions, and analysis of covariance (ANCOVA) to compare the study groups. The ANCOVA analyses were adjusted for age. For follow-up, proportionality requirements were met, and we used multivariable Cox hazards regression modelling to compare 10-year and 18-year mortality in various prefrailty/frailty and multimorbidity groups and calculated hazard ratios (HR) with 95% confidence intervals (CI). Schoenfeld residuals were inspected to confirm proportional hazards assumption. The group with no prefrailty/frailty and no multimorbidity was used as the reference category and variables were used for adjustments as follows: Age (continuous variable) in model 1; besides age variables significantly different between reference category and multimorbidity in model 2 (BMI, memory disturbance, regular drug use); besides age variables significantly different between reference category and frailty in model 3 (BMI, smoking years, memory disturbance, mental health, ADL disability, regular drug use). Model 3 was also the final model because it included all previous variables for adjustment. Although mobility disability in men with frailty was significantly different from reference category, it was not used in the analyses because of overlap with the definition of the frailty phenotype definition. To estimate selection bias, we also compared mortality among those men who were alive in 2000 but did not respond to the questionnaire survey, as well as those who responded but whose frailty/multimorbidity status in 2000 could not be assessed due to incomplete data. The role of attrition bias was assessed to be negligible, because the outcome (total mortality during follow-up) was retrieved from a reliable country-wide register.

In all analyses, two-sided *P* values < 0.05 were taken to denote statistical significance. Statistical analyses were performed using NCSS statistical software (Kaysville, UT, www.ncss.com, version 8).

### Ethical statement

The follow-up complies with the Declaration of Helsinki, the research protocol has been approved by the ethical committee of the Department of Medicine, Helsinki University Central Hospital, and informed consent has been obtained from the subjects. The study is registered as ClinicalTrials.gov identifier: NCT02526082.

## Results

**Fig. 1:**
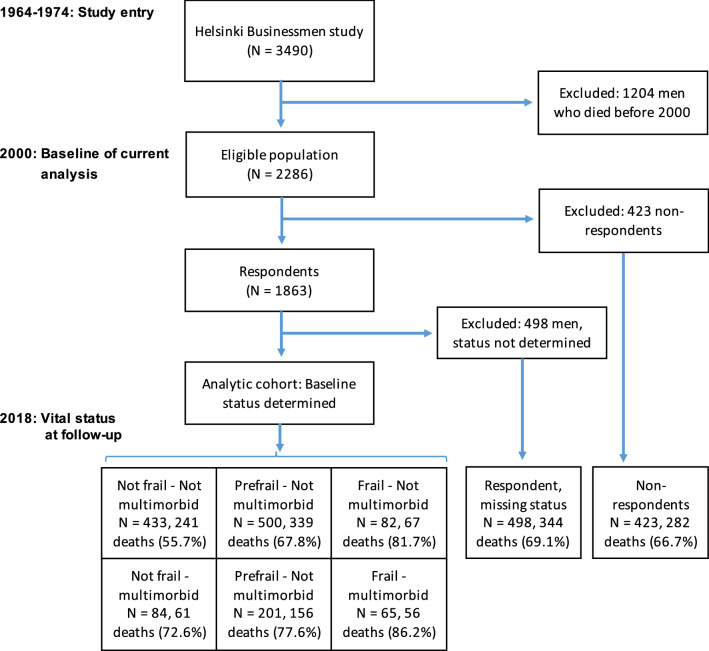
Flow chart of the study

In 2000, the questionnaire was returned by 1863 men (81.5% of 2286 survivors), of them 98% were community-living with an age range from 66 to 81 years (median 74 years). Due to incomplete data in questionnaires, frailty phenotype and multimorbidity status could not be assessed in 498 men. Thus, the analytical sample consisted of 1365 men, of them 99% were community-living with an age range from 66 to 81 years (median 73 years). However, 18-year mortality follow-up was complete for all 2286 survivors in 2000. The flow chart of the study is shown in Fig. 1 and baseline data of various study groups are compared in Table 1. In the analytic sample, prevalencies of multimorbidity, prefrailty and frailty phenotype were 25.6% (*n* = 350), 51.4% (*n* = 701) and 10.8% (*n* = 147), respectively, while both mobility disability (6.1%, *n* = 83) and ADL disability (2.2%, *n* = 30) were less frequent.

**Table 1:** Baseline characteristics of the study groups

Variable^a^	Status at baseline						*P* value between groups
	No prefrailty/frailty, no multimorbidity, *n* = 433	Prefrailty without multimorbidity, *n* = 500	Frailty without multimorbidity *n* = 82	Multimorbidity without frailty, *n* = 84	Multimorbidity with prefrailty, *n* = 201	Multimorbidity with frailty, *n* = 65	
Age in 2000, y, median (IQR)	71 (69–75)	73 (70–76)	75 (71–78)	72 (70–76)	74 (71–78)	76 (71–79)	< 0.001
BMI, kg/m^2^	25.9 (0.1)	25.6 (0.1)	24.8 (0.3)^b^	26.7 (0.3)^c^	26.2 (0.2)	25.4 (0.4)	< 0.001
BMI change since midlife, kg/m^2^	+ 0.7 (0.1)	− 0.01 (0.1)	− 1.3 (0.3)^b^	+ 0.9 (0.3)	+ 0.1 (0.2)	− 1.2 (0.3)	< 0.001
Smokers, *n* = 110, %	4.8	9.4	14.6^b^	4.8	9.5	10.8	0.01
Smoking years	13.2 (0.9)	17.3 (0.8)	18.7 (2.2)^b^	14.2 (1.7)	18.4 (1.2)	18.6 (2.0)	0.001
Alcohol, g/week	110.5 (7.2)	128.1 (6.6)	148.5 (16.7)	130.0 (16.3)	123.2 (10.5)	144.0 (18.9)	0.21
Living alone (widowed/divorced/unmarried), *n* = 146, %	8.6	12.1	11.7	14.4	12.4	18.6	0.16
Mental Health^d^, score	89.2 (0.1)	80.0 (0.8)	63.5 (2.1) ^b^	87.5 (1.6)	75.5 (1.1)	62.1 (1.9)	< 0.001 (ln transformed
Mental Health^d^ score < 50, *n* = 91, %	0.8	7.0	28.8	0	12.2	22.5	< 0.001
Subjective memory disturbance, *n* = 206, %	7.8	14.6	21.7 ^b^	17.1	25.8	44.6	< 0.001
ADL disability, *n* = 30, %	0	1.6	10.1 ^b^	0	1.5	17.2	< 0.001
Mobility disability, *n* = 83, %	0.2	3.0	27.8 ^b^	2.4 ^c^	7.6	43.1	< 0.001
Without regular medication, *n* = 240, %	27.7	19.4	6.1 ^b^	11.9 ^c^	4.0	0	< 0.001
Without chronic disease^e^, *n* = 617, %	63.3	60.8	47.6	0	0	0	< 0.001
Hypertension, *n* = 507, %	39.1	36.9	37.3	38.1	45.2	54.7	0.07
Diabetes, *n* = 116, %	4.8	4.4	8.3	17.9	21.4	21.5	< 0.001
Cancer, *n* = 173, %	8.8	9.7	14.9	22.6	25.9	21.5	< 0.001
Musculoskeletal disorder, *n* = 338, %	14.2	16.4	30.1	50.0	51.7	66.2	< 0.001
Chronic pulmonary disease, *n* = 103, %	3.1	4.7	9.0	19.0	16.4	24.6	< 0.001
CHD, *n* = 273, %	11.6	10.9	21.1	50.0	47.8	40.0	< 0.001
Stroke, *n* = 150, %	3.9	9.8	10.0	17.9	22.9	36.9	< 0.001
Peripheral artery disease, *n* = 183, %	4.3	7.6	17.1	26.2	34.3	46.2	< 0.001
Heart failure, *n* = 168, %	4.4	6.0	15.2	25.0	33.8	40.0	< 0.001

*ADL* activities of daily living; *BMI* body mass index; *CHD* coronary heart disease; *IQR* interquartile range

^a^Continuous variables are age adjusted, mean (SE)

^b^ and ^c^mark those variables significantly different from the group with no prefrailty/frailty and no multimorbidity and which were used for adjustments in Cox regression analyses as described in Methods

^d^Mental Health domain of RAND-36

^e^Includes any of the 8 chronic diseases defined in Methods

Of the whole sample, 433 (31.7%) were nonfrail without multimorbity, 500 (36.6%) and 82 (6.0%) men had prefrailty or frailty, respectively, without multimorbidity; 84 (6.2%) men had multimorbidity only, and 201 (14.7%) and 65 (4.8%) men had prefrailty or frailty together with multimorbidity. Multimorbidity was mainly caused by cardiovascular and pulmonary diseases, cancer and diabetes, but musculoskeletal disorders were common also in men with frailty. The prevalence of hypertension was common and not statistically significantly different between the groups.

**Fig. 2:**
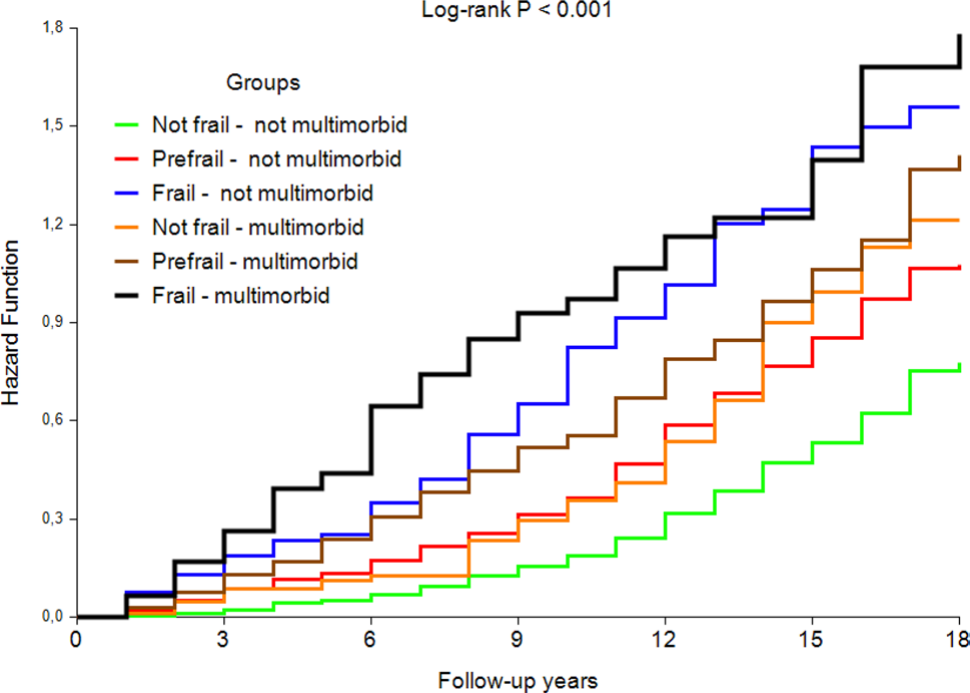
Cumulative 18-year total mortality in the study groups according to phenotypic frailty and multimorbidity status

During the 18-year follow-up, 920 men died (67.4%). Unadjusted Kaplan-Maier curves for 18-year mortality in the study groups show the marked excess mortality associated with frailty phenotype with or without multimorbidity (Fig. 2). These observations were confirmed in the adjusted Cox analyses using men with no prefrailty/frailty and no multimorbidity as the reference category (Table 2).

**Table 2:** Serially adjusted Cox regression for 18-year mortality according to baseline frailty and multimorbidity status

Model	Hazard ratio (95% confidence interval) for 18-year total mortality					
	No prefrailty/frailtyno multimorbidity, *n* = 433	Prefrailty without multimorbidity, *n* = 500	Frailty without multimorbidity, *n* = 82	Multimorbidity without frailty, *n* = 84	Multimorbidity with prefrailty, *n* = 201	Multimorbidity with frailty, *n* = 65
Unadjusted	1.0 (reference)	1.50 (1.24–1.78)	2.19 (1.58–3.03)	1.83 (1.40–2.38)	2.15 (1.75–2.64)	3.53 (2.66–4.68)
Model 1	1.0 (reference)	1.33 (1.11–1.60)	1.77 (1.27–2.46)	1.66 (1.27–2.17)	1.74 (1.42–2.14)	3.14 (2.36–4.18)
Model 2	1.0 (reference)	1.32 (1.11–1.55)	1.79 (1.39–2.51)	1.59 (1.12–1.98)	1.73 (1.35–2.04)	3.05 (1.60–3.10)
Model 3	1.0 (reference)	1.24 (1.02–1.50)	1.62 (1.13–2.31)	1.55 (1.17–2.06)	1.61 (1.28–2.02)	2.93 (2.10–4.07)

Model 1: adjusted for age (continuous variable); model 2: besides age adjusted for variables significantly different between reference category and multimorbidity (BMI, memory disturbance, regular drug use); model 3 = final model: besides age adjusted for variables significantly different between reference category and frailty (BMI, smoking years, memory disturbance, mental health, ADL disability, regular drug use)

*ADL* activities of daily living; *BMI* body mass index

In the final model frailty phenotype (HR 1.61, 95% CI 1.13–2.31) alone without multimorbidity was associated with similar mortality risk to multimorbidity alone (HR 1.55, 95% CI 1.17–2.06), also prefrailty alone was significant (HR 1.24, 95% CI 1.02–1.50).The presence of both frailty and multimorbidity indicated a strong mortality risk (HR 2.93, 95% CI 2.10–4.07).

In order to further separate a possible confounder, disability, from analyses, we rerun the fully adjusted analysis after excluding men with either ADL disability or mobility disability (*n* = 98). No prefrailty/frailty and no multimorbidity group as reference, the HRs (with 95% CI) were 1.24 (1.10–1.50), 1.57 (1.03–2.38), 1.57 (1.18–2.10), 1.56 (1.23–1.98), and 2.74 (1.86–4.03) for the prefrail or frail without multimorbidity, multimorbidity alone, and multimorbidity with prefrailty or frailty, respectively. We also analysed 18-year mortality among non-respondents (*n* = 423) and among those whose frailty/multimorbidity status could not be assessed in 2000 (*n* = 498). Mortality in these groups was similar to the average of the analytic sample (Fig. 1).

Finally, because the 18-year follow-up is very long for septuagenarian men, we also analysed HRs during the first 10 years or follow-up. In the final model with no prefrailty/frailty and no multimorbidity group as reference, the HRs (with 95% CI) were 1.42 (1.01–2.00), 1.99 (1.15–3.46), 1.78 (1.10–2.89), 2.53 (1.75–3.65), and 4.68 (2.94 to 7.43) for the prefrail or frail without multimorbidity, multimorbidity alone, and multimorbidity with prefrailty or frailty, respectively.

## Discussion

Our 18-year follow-up suggests impact of both prefrailty and frailty phenotype as defined in this study on mortality risk in 70 + , community-living men, independently of chronic, clinically meaningful diseases or disability. Especially the presence of both multimorbidity and frailty phenotype was a very strong indicator of mortality risk. Although men with frailty had more memory complaints and worse general psychological health than men without frailty, the mortality results remained significant after various adjustments, also with variables related to mental and cognitive state.

### Strengths and limitations

The strengths of the study include its long follow-up and reliable assessment of mortality from national registers. The response rate was also satisfactory and analyses of the mortality among non-respondents suggest that a major bias is unlikely. A further strength of this homogenous male population from the highest social strata is that socioeconomic factors are not likely to affect the results. Hazard ratios of mortality were relatively insensitive to several adjustments suggesting that residual confounding is not likely.

At the same time, the homogeneity is also an obvious limitation for generalisability. Furthermore, the cohort mainly represents functional older men, because almost all were home-dwelling and assessed proportions of ADL- or mobility disability were only 2% and 6%, respectively.

Long follow-up is also a limitation, because clinical status was defined at baseline and potential changes in frailty and morbidity status during follow-up is not known. This may lead to underestimation of relationships with mortality, which is supported by higher HRs during the 10-year vs 18-year follow-up.

Self-report of diseases is a limitation, but a recent analysis suggested that self-report of many chronic conditions can provide a reliable estimate [24], and comparison of the disease prevalencies with national statistics largely supports this. Moreover, reliability is probably better in this type of cohort with high social status.

Currently there is no consensus definition of frailty, and various modifications of the CHS frailty phenotype [8] have been used in different studies. For our modification, prognostic validation has been shown earlier [21]. Moreover, questionnaire data without clinical strength measurement have also been used to define frailty in other cohorts. The simplified Women's Health Initiative (sWHI) frailty score, for example, showed high correlations with the standard CHS frailty phenotype [25]. Whether weight loss in our cohort was intentional or unintentional is not known.

Assessment of cognitive and mental status were also based on self-report. However, the RAND-36 Mental Health domain has been used to determine the presence of general psychological disorder [26], and its score was significantly lower among those who reported mental disorder. Although self-reported memory disturbance alone is not sufficient for general screening of dementia, its presence in 2000 significantly predicted incident dementia during a 14-year follow-up (unpublished observation, types of dementia were certified from death certificates as described in Ref. [27]).

A final limitation is that in an observational study causal inferences cannot be made.

### Comparison to previous studies

Multimorbidity has received a lot of attention during the last decade for several reasons. It has suggested to be more than the sum of its parts, it leads to polypharmacy and complicated care with possibilities of adverse effects, and may predispose to intervening diseases like infections. Accordingly, it is considered to be a major burden for healthcare in ageing societies. Because various definitions of multimorbidity can also include conditions of less serious nature [22], in the present study we chose to include only clinically meaningful chronic diseases, such as major cardiovascular- and cerebrovascular disease, diabetes, chronic pulmonary disease, cancer, and musculoskeletal disease including rheumatic diseases and osteoarthritis.

Disability has been shown to exert a higher mortality risk than multimorbidity in an older population with frequent (32%) prevalence of disability [16]. In the present study we specifically wished to evaluate the independent and clinically meaningful effect of the concept of frailty over traditional diseases and also disabilities on prognosis. Therefore, we defined frailty as a phenotype, not as cumulative deficits which usually include also diagnosed diseases and disabilities. In addition, we assessed the impact of prefrailty, which is a less homogenous and more controversial condition and with a potential of reversal to non-frailty [28]. Our findings from this long follow-up show that prefrailty alone has prognostic significance, suggesting that it could be considered as an earlier target of prevention. Indeed, it is surprising that according to cumulative hazard function the effect of prefrailty on mortality appears to be similar to multimorbidity at least for the first 12–15 years of follow-up (Fig. 2). The combination of prefrailty and multimorbidity conferred a stronger mortality risk.

What may be the reason for quite moderate effect of multimorbidity (without frailty) on mortality prognosis in our cohort? Many of the diseases included are nevertheless frequent causes of death. The participants had high socioeconomic status and can be seen as a population subgroup nested within the Finnish population at large. They may represent a group which is likely to be treated early, to have high adherence to treatments and a better prognosis for chronic conditions than the general population. For example, almost half of the of the responding men were using statins in the 2015 survey when median age was 85 years [29], and statin treatment has been shown to improve prognosis also in old age [30]. On the other hand, frailty alone does not necessarily lead to medical treatments, although it is possible that frail men have subclinical disease worsening their prognosis. Midlife CVD risk factors have been shown to associate with development of frailty [31]. In geriatrics, polypharmacy is often seen as a general threat, but further research is clearly warranted of the effects of specific drugs on prognosis. We are presently conducting detailed analyses of long-term drug effects in the HBS cohort.

To conclude: Although multimorbidity is generally considered a substantial health problem, our long-term observational study emphasises that frailty phenotype alone may confer a higher risk, and a combination of multimorbidity and frailty is an especially strong predictor of mortality. The results highlight identification of frailty and potentially also prefrailty as a clinically meaningful and feasible method to more effectively assess patient prognosis at older ages.
